# Acceptability and Feasibility of “Village,” a Digital Communication App for Young People Experiencing Low Mood, Thoughts of Self-harm, and Suicidal Ideation to Obtain Support From Family and Friends: Mixed Methods Pilot Open Trial

**DOI:** 10.2196/41273

**Published:** 2023-03-13

**Authors:** Hiran Thabrew, Harshali Kumar, Evandah Steadman

**Affiliations:** 1 Department of Psychological Medicine The University go Auckland Auckland New Zealand; 2 N/A Auckland New Zealand

**Keywords:** youth, suicide, self-harm, depression, support, application, mobile phone

## Abstract

**Background:**

Young people experiencing low mood, thoughts related to self-harm, and suicidal ideation often struggle to communicate their emotions and receive timely support from family and friends. Technologically delivered support interventions may be useful in addressing this need.

**Objective:**

This paper aimed to evaluate the acceptability and feasibility of “Village,” a communication app co-designed with young people and their family and friends from New Zealand.

**Methods:**

A mixed methods pilot open trial design was adopted. Participants were primarily recruited via social media advertisements and clinicians in specialist mental health services over an 8-month period. The primary outcomes were acceptability of the app (via thematically analyzed qualitative feedback and retention rates) and feasibility of conducting a larger randomized controlled trial gauged via effectiveness of recruitment methods, completion of chosen outcome measures, and occurrence of unanticipated operational issues. Secondary outcomes were app usability, safety, and changes in symptoms of depression (via the Patient Health Questionnaire–9 modified for adolescents), suicidal ideation (on the Suicidal Ideation Questionnaire), and functioning (using the World Health Organization Disability Assessment Schedule 2.0 or Child and Youth version).

**Results:**

A total of 26 young people (“users”) were enrolled in the trial, of which 21 recruited friends and family members (“buddies”) and completed quantitative outcome measures at baseline, 4 weeks, and 3 months. Furthermore, 13 users and 12 buddies also provided qualitative feedback about the app, identifying the key themes of appeal of app features and layout, usefulness of its content, and technological challenges (primarily with onboarding and notifications). Users gave Village a mean rating of 3.8 (range 2.7-4.6) out of 5 on a 5-point scale for app quality and an overall star rating of 3.4 out of 5 for subjective quality. Within this limited sample, users reported a clinically significant reduction in depressive symptoms (*P*=.007), but nonsignificant changes in suicidal ideation and functioning. The embedded risk detection software was activated on 3 occasions, and no additional support was required for users.

**Conclusions:**

During this open trial, Village was found to be acceptable, usable, and safe. The feasibility of a larger randomized controlled trial was also confirmed after some modifications to the recruitment strategy and app.

**Trial Registration:**

Australian New Zealand Clinical Trials Network Registry ACTRN12620000241932p; https://tinyurl.com/ya6t4fx2

## Introduction

### Background

Internationally, rates of mental distress and depression among young people have steadily increased over the past few decades [1-3]. In the wake of COVID-19, they are predicted to rise further as part of a “long psychosocial tail” of pandemic-related consequences [4,5]. Young people experiencing these issues often struggle to verbally express strong and fluctuating emotions and reach out to their families or peers for timely assistance [6,7]. Instead, they may resort to the use of maladaptive coping strategies, including impulsive or planned self-harm [8]. Self-harm is defined as intentional self-injury or self-poisoning, irrespective of the extent of suicidal intent [9]. In New Zealand, approximately 24% of high school students were reported to be engaging in self-harm (17.9% of males and 29.1% of females aged 13-19 years) [10]. The consequences of self-harm include hospitalization (80.8 per 100,000 males and 212 per 100,000 females aged 15-19 years) [11] and suicide [12]. Currently, owing to a likely combination of social and family-related factors [12], New Zealand holds the dubious honor of having the highest rate of suicide among young people in the developed world (19.3 per 100,000 young people and even higher—36.4 per 100,000 among Indigenous Māori young people) [13].

Over the past 20 years, rapidly evolving smart technology has led to the development of a range of digital health (eHealth) interventions, including those specifically designed to improve mental health [14]. These include information-oriented websites for learning about stress management, game-based therapies on the web for common mental health problems, and mobile health self-help apps to improve well-being [15]. Such interventions offer particular appeal to young people who have grown up as “digital natives” [16]. Some have been shown to be as effective as face-to-face therapies [17], and as such, are now recommended by the National Institute for Clinical Excellence in the United Kingdom as a first-line treatment for adolescent anxiety and depression [18] and by international groups such as the Lancet Global Mental Health Group [19] as a scalable means of addressing common mental health issues. Purported advantages of eHealth interventions include cost-effectiveness, flexibility of use, and potential for increasing equity of treatment access and reducing stigma [20]. These are reflected in people’s keenness to use eHealth interventions [21]. Recent reviews have confirmed the benefits of mobile health interventions in improving well-being and suicide-related psychosocial outcomes [22,23].

Alongside these substantial technological developments, there has been a growing awareness that self-empowerment of well-being [24], family-inclusive approaches to health [25], and peer support [26] can improve the quality of care and outcomes for people experiencing mental health issues. Empowering people to manage their own well-being in the first instance is encouraged by consumer organizations and the World Health Organization 2013-2020 Mental Health Action Plan [27]. Family-inclusive approaches have also been shown to improve resilience among young people [28]. Recent evidence from King et al [29] in the United Kingdom suggests that, despite the lackluster performance of widely used suicide risk management and scoring systems, youth-nominated support teams (consisting of peers and family members) can actually be effective in reducing rates of suicidal ideation in the short term and rates of suicide among young people for over a decade [30]. The key ingredients identified from this relatively novel approach are social support, suicide prevention literacy, and adolescent skill development (learning to communicate and accept help from others) [30]. To date, such support has not been tested using eHealth approaches.

With these issues in mind, in 2020, our team undertook a co-design process to develop a working prototype for a digital, youth-nominated support system that might benefit young people experiencing low mood, self-harm, and suicidal ideation. Approximately 40 New Zealand youth (including many who had experienced these issues or were affiliated with a national telephone-based support organization [Youthline]), 20 family members, 3 mental health clinicians, and a team of 6 IT specialists from Datacom, one of New Zealand’s largest IT companies, were involved in the app’s co-design. After a 6-month, agile, iterative process that included *sprints* of app development and “scrums” of product review [31], “Village,” an innovative communication app (ie, an app primarily focused on improving communication between sets of users) was developed for pilot evaluation. Mental health–related content was initially drafted by the primary investigator (a child and adolescent psychiatrist) and refined after consultation with other mental health clinicians and user feedback. The purpose of Village is to help users (young people) obtain regular and timely support from a network of buddies (family members, whanau, or peers), who are in turn educated and supported to respond appropriately to their messages. During the co-design process, younger adolescents voiced a preference for receiving support from family members, whereas older adolescents and adults stated that they would prefer support via peers. As such, the app was consciously developed to meet the needs of adolescent and adult users as well as those of adolescent and adult supporters. The design of Village is underpinned by four theoretical constructs:

1. The nonviolent communication model helps people recognize their specific emotions and needs before they engage in self-empathy or any kind of conflict or problem-solving involving other people. Once self-awareness is gained, it teaches people to make requests of others that are specific and free of demand [32].
2. Systems theory is a philosophy that focuses on the interdependence of individuals in a group to help understand and optimize the achievements of the system [33]. It also aligns with New Zealand Māori concept of “Whanau Ora,” which identifies connection with family (“whanau”) as an integral part of good health [34].
3. Supportive therapy is designed to reduce psychological conflict and strengthen a person’s defenses through the use of various techniques such as reassurance, suggestion, counseling, and education [35].
4. Strength-based therapy seeks to focus a person’s attention on their own strengths, resourcefulness, and resilience to help them recognize positive aspects of their identity and to use these qualities to move forward [36].

### Objective

The primary aim of this open trial was to evaluate the acceptability and usability of the prototype app. Second, we sought to undertake a preliminary evaluation of the app’s usability, safety, and efficacy in altering mood, suicidal ideation, and functioning, as well as the feasibility of undertaking a more definitive randomized controlled trial (RCT). The key hypotheses were that the Village app would be acceptable, usable, and safe for users and buddies; that its use would lead to an improvement in mood and functioning and a reduction in suicidal ideation; and that an RCT using chosen or modified outcome measures and parameters would be feasible.

## Methods

### Study Design

The single-arm, open trial of Village was conceptualized by 2 authors (HT and ES), and a mixed methods design was used. Participants were initially recruited by 2 authors (HK and HT) via specialist child and adolescent mental health services and primary health services in Auckland, New Zealand. Owing to low enrollment rates between November 2020 and February 2021 (most likely because of COVID-19–related service disruption), after the amendment of ethics approval, recruitment was expanded to social media advertisements (Facebook and Instagram linked to REDCap [Research Electronic Data Capture; Vanderbilt University], which recorded the number of individuals who signed up) between March 2021 and July 2021. Participants were selected by convenience and were supported to download the app via written and verbal instructions at the time of recruitment. They were encouraged to use the app as they wished to (with no set frequency) for 4 weeks. They were prompted (via automated REDCap messages and text if there was no response within 48 hours) to complete web-based quantitative outcome measures via REDCap at 3 time points: baseline, 4 weeks, and 3 months. After the use of Village for 4 weeks, more in-depth qualitative information regarding user experiences and feedback from buddies was collected by 1 author (HK, a female research assistant with prior experience of qualitative research and no prior relationship with participants before the study) on a single occasion, in person (one-on-one), at university premises via individual semistructured interviews lasting for 30-60 minutes. The interview schedule was developed by the research team and was road-tested with a couple of young people and adults. Interviewees were informed that questions were being asked to evaluate and improve the app before it became publicly available. The interviews were audio-recorded and transcribed by a certified transcriber who signed a confidentiality agreement. Data analysis and interpretation were undertaken by 2 authors (HT and HK). The paper was drafted by the first author (HT) and reviewed by all the authors (HT, HK, and ES) before submission.

### Study Population

Young people, aged between 16 and 25 years and of any gender, were invited to participate in the study. Eligible participants were experiencing low mood (self-reported, no quantified cutoff score), self-harm, or suicidal ideation; receiving psychotherapy or medication treatment; spoke adequate English to use the app; had access to a smartphone (iPhone or Android mobile); were able to provide electronic or written informed consent; and were able to nominate at least one buddy aged >16 years (as agreed with the ethics committee). Individuals who did not meet these criteria or who were currently receiving dialectical behavioral therapy (DBT), which includes active family and therapist support, were excluded. Each young person identified one of their friends to be invited to participate in follow-up interviews. These individuals provided separate written consent and demographic information before being interviewed.

### Intervention

**Figure 1:**
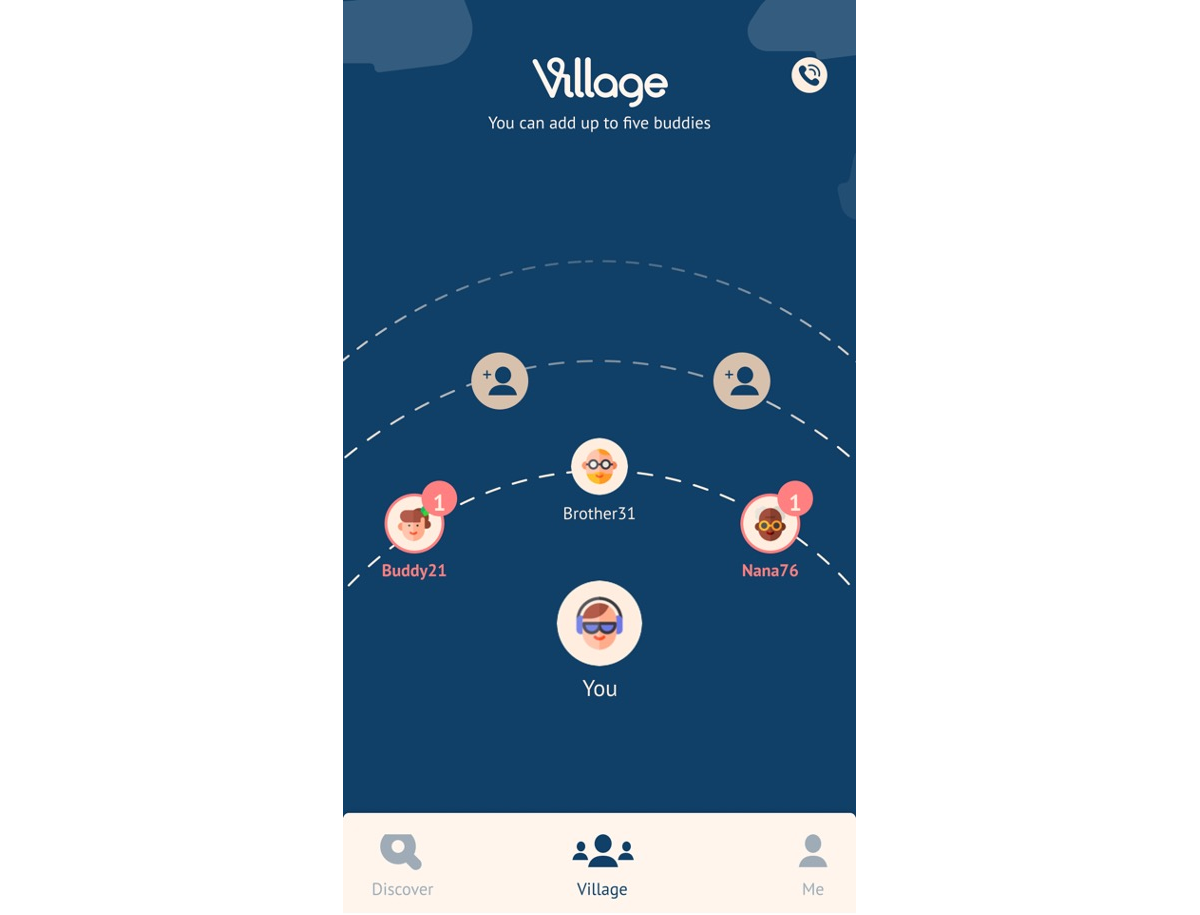
Home screen.

**Figure 2:**
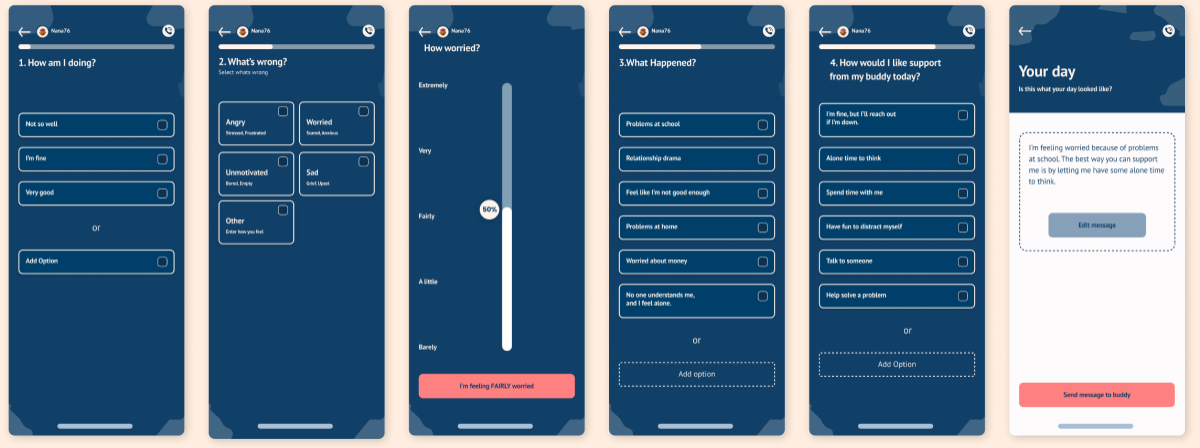
Supported messages. Higher-resolution version of this figure is available in Multimedia Appendix 1.

**Figure 3:**
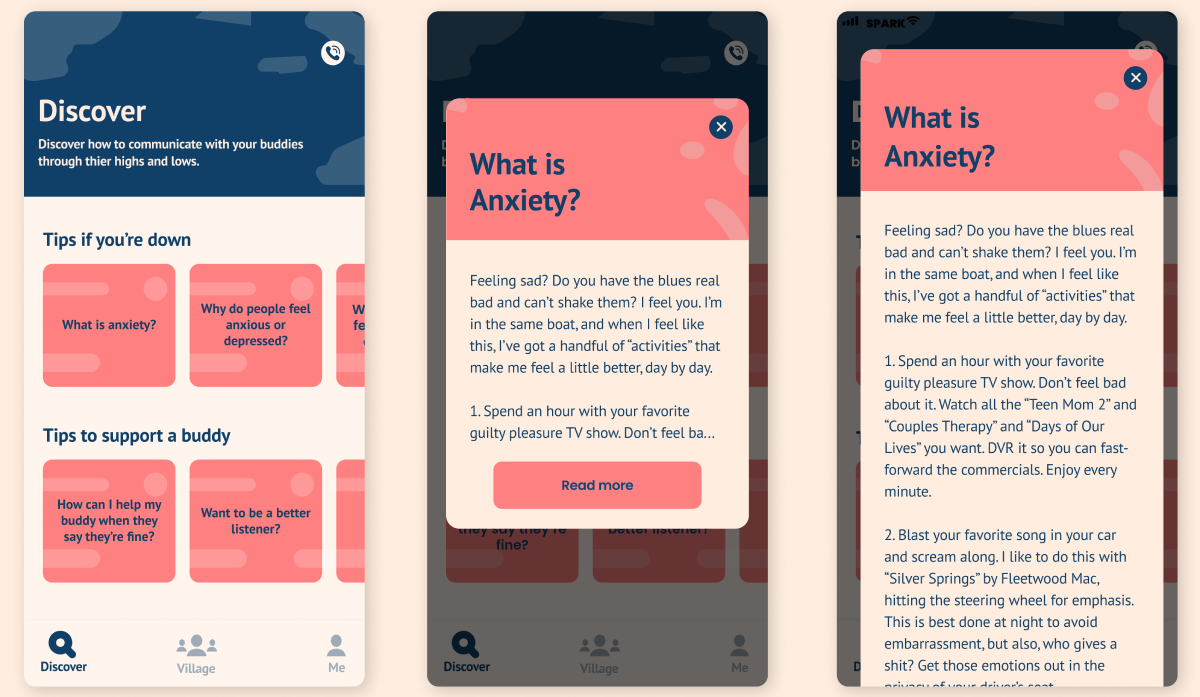
Example of information module for buddies.

Village is a communication app that helps young people experiencing low mood, self-harm, or suicidal ideation connect with a self-nominated support network of peers or family members. Young people (“users”) nominate and, via a text link, invite between 1 and 5 support people (“buddies”), to whom they can either send (the same or different) messages as often as they want and from whom they can receive daily check-ins and support. Users and buddies receive instructions on how to use the app through a series of onboarding messages. Users are guided to create messages letting buddies know how they are feeling and why, as well as what support they would ideally like them to provide. Buddies are coached via the app to respond sensitively to user messages. They were also provided with information about communication, common mental health issues (such as anxiety, depression, and self-harm), and what to do if they were worried about users’ safety. No additional or external support was provided to buddies during the trial. Software built into Village detects “risky messages” based on previously proven keywords for detecting high risk adolescents’ behavior on the web such as “suicide,” “kill myself,” “end my life,” “useless,” “want to die,” “dead,” and “worthless” [37]. This software alerts users and gives them the option of seeking immediate help via a national telephone helpline or during the trial, being contacted by a member of the research team (HT) within 24 hours. All data were stored on user (and buddy) devices and erased when the app was deleted. For privacy reasons, no data were sent to external servers. Screenshots of Village are presented in Figures 1-3.

### Outcome Measures

*Acceptability* of Village was qualitatively evaluated via thematically analyzed feedback from user and buddy interviews and retention rates after enrollment. The *Feasibility* of conducting an RCT was evaluated based on the proportion and speed of recruitment via different means, completion of chosen outcome measures, and occurrence of unanticipated operational issues. *Usability* was quantitatively evaluated via the user version of the Mobile Application Rating Scale (uMARS) [38] and qualitatively evaluated via feedback from user and buddy interviews (see Multimedia Appendix 2 for semistructured interview questions). The uMARS is a validated and reliable self-reported scale that consists of 6 sections (app engagement, functionality, esthetics, information, subjective impact, and perceived impact) and 3 subscales (overall app quality, subjective quality, and perceived impact). The uMARS questionnaire has a high internal consistency (Cronbach α=.90) and good test-retest reliability for the total scale. *Safety* was evaluated based on the frequency and outcome of risk detection software activation and self-reported episodes of self-harm or hospitalization during the follow-up interviews. *Efficacy* was evaluated by measuring changes over time in symptoms of major depressive disorder using the Patient Health Questionnaire–9 modified for adolescents [39], suicidal ideation using the Suicidal Ideation Questionnaire (SIQ) [40], and functioning using the World Health Organization Disability Assessment Schedule (WHODAS) 2.0 for participants aged >18 years and WHODAS–Children and Youth (WHODAS-CY) for participants aged <18 year [41,42]. Further details of the outcome measurement schedule are presented in Table 1. The Patient Health Questionnaire–9 is a 9-item questionnaire that assesses the degree of depression severity and has moderate internal consistency (Cronbach α=.7−.93) and good internal reliability (Cronbach α=.89). The level of severity score included ranges from 0-4 (minimal), 5-9 (mild), 10-14 (moderate), 15-19 (moderately severe), and 20-27 (severe). The SIQ is a 30-item questionnaire that measures the frequency of suicidal thoughts among adolescents. It has high internal consistency (Cronbach α=.97) and test-retest reliability (Cronbach α=.86). A score of ≥41 indicates that the individual requires further evaluation of the psychopathology and suicide risk. The WHODAS 2.0 is a 36-item scale that consists of six domains that measure the level of functioning of an adult in the last 30 days within the following areas: (1) cognition, (2) mobility, (3) self-care, (4) getting along, (5) life activities, and (6) participation. The scale has high internal consistency (Cronbach α=.98) and test-retest reliability (Cronbach α=.92). The WHODAS-CY has also been shown to have a high internal reliability (Cronbach α=.84). It measures all areas of functioning as per the WHODAS 2.0 and includes a section regarding school. Both WHODAS measures contain the same score ranging from 0 to 100 (0=no disability and 100=full disability).

**Table 1:** Schedule of outcome measurement.

Measures	Baseline	4-week follow-up	3-month follow-up
PHQ-A^a^	✓^b^	✓	✓
SIQ^c^	✓	✓	✓
WHODAS 2.0^d^	✓	✓	✓
WHODAS-CY^e^	✓	✓	✓
uMARS^f^		✓	
Semistructured interviews		✓	

^a^PHQ-A: Patient Health Questionnaire–9 modified for adolescents.

^b^✓: denotes completion of measures.

^c^SIQ: Suicidal Ideation Questionnaire.

^d^WHODAS 2.0: World Health Organization Disability Assessment Schedule 2.0.

^e^WHODAS-CY: World Health Organization Disability Assessment Schedule–Children and Youth.

^f^uMARS: user version of the Mobile Application Rating Scale.

### Data Analysis and Statistical Methodology

*Quantitative data* were analyzed by 2 authors (HK and HT) using Microsoft Excel (version 16.52) and SPSS (version 27.0, IBM Corp). Analyses included basic descriptive statistics for demographic characteristics and changes in depression, suicidal ideation, and functioning. Linear mixed models were used to assess changes in depression, suicidal ideation, and functioning over time (between baseline, 4-week, and 12-week follow-up), accounting for repeated within-person measures over the study period. This analysis enabled the inclusion of information from individuals who did not complete or did not have data at all the 3 time points. *P* values of <.05 were considered statistically significant and 95% CIs were presented. *Qualitative data* from semistructured interviews of users and buddies were analyzed by 2 authors (HT and HK) using NVivo (QSR International) software and the 6-stage method of thematic analysis by Braun and Clarke [43] (familiarization, coding, generating themes from the data, reviewing themes, defining and naming themes, and writing up). The transcripts were not returned to participants for review. Key themes, subthemes, and supporting quotes were identified and coding discrepancies, if any, were addressed by consensus.

### Ethics Approval

This study was approved by the New Zealand Health and Disability Ethics Committee (20/NTB/116). Users recruited via clinical service recruits and interviewed buddies provided written consent, and users recruited through the web provided electronic consent via REDCap. Written data were stored in secure filing cabinets, and electronic data were stored on a secure server, as per the University of Auckland regulations. A privacy impact assessment conducted at the request of the ethics committee rated the study as “low risk.” Although users had the option of being put through a telephone helpline if risky messages were detected by the app, none of their information was forwarded to any external agencies. All participants were encouraged to disclose any potential adverse events, including self-harm, suicide attempts, and hospitalization, to the research team at any stage of the study, and they were specifically asked about these events during the follow-up interviews. A log of these events and the number, dates, and outcomes of risky messages were maintained by the research team. Owing to the nature of the study, no external data safety monitoring committee was used. App users were informed that they were free to withdraw from the study at any stage. Regardless of the duration of the participants’ involvement, all enrolled users and buddies received a NZ $50 (US $30) gift voucher on exit from the study.

## Results

### Participant Characteristics

A total of 321 young people were made aware of the study, 23 via child and adolescent mental health services, 5 via general practice services, and 293 via social media advertisements. A total of 14 clinicians were involved in referring young people from the services mentioned above. Of those who knew about the study, 156 young people did not meet the eligibility criteria; 78 did not respond to contact from the research team about receiving more information regarding the trial; 58 declined to participate because they were not interested, their mental health was stable, or they did not have a suitable buddy; and 8 agreed to participate, but did not complete baseline measures and did not download the app. This resulted in 26 users, 21 of whom managed to enroll ≥1 buddies and complete the trial. The demographic characteristics of both groups are presented in Table 2. Unfortunately, because the REDCap forms are not mandatory, not all outcome measures were completed at all time points. At 12 weeks, all users of Village and their nominated buddies were invited to be interviewed about their experiences with the app. Of these, 13 users and 12 buddies agreed to participate.

**Table 2:** Participant characteristics.

Characteristic				Value
**Participants^a^, n (%)**				
	**Young people or users (n=26)**			
		Registered and completed baseline outcome measures	26 (100)	
		Completed 4 and 12-week outcome measures	21 (81)	
		Completed follow-up interviews	13 (50)	
	**Buddies (n=21)**			
		Registered	21 (100)	
		Completed follow-up interviews	12 (57)	
**Age (years), mean (range)**				
	Young people or users			17.7 (16-25)
	Buddies			23.6 (16-53)
**Gender, n (%)**				
	**Young people or users (n=26)**			
		Female	17 (65)	
		Male	6 (23)	
		Nonbinary	3 (12)	
	**Buddies (n=21)**			
		Female	12 (57)	
		Male	5 (24)	
		Nonbinary	2 (10)	
		Preferred not to answer	1 (5)	
**Ethnicity, n (%)**				
	**Young people or users (n=26)**			
		Māori	4 (15)	
		New Zealand European	17 (65)	
		Asian	4 (15)	
		MELAA^b^	1 (4)	
	**Buddies (n=21)**			
		Māori	2 (10)	
		New Zealand European	12 (57)	
		Asian	2 (10)	
		MELAA	1 (5)	
		Other^c^	4 (19)	
**Reason for participation and relationship to young person^d^, n (%)**				
	**Young people or users (n=26)**			
		Low mood	23 (88)	
		Self-harm	9 (35)	
		Suicidal ideation	13 (50)	
		Other	7 (27)	
	**Buddies (n=21)**			
		Parents	3 (14)	
		Siblings	1 (5)	
		Partners	2 (10)	
		Friends	14 (67)	
**Place of recruitment, n (%)**				
	**Young people or users (n=26)**			
		Child and adolescent mental health service	8 (31)	
		General practice	4 (15)	
		Social media	14 (54)	
	**Buddies**			
		Invited by a young person	—^e^	

^a^One buddy did not complete the demographic form.

^b^MELAA: Middle Eastern, Latin American, and African.

^c^Other: Fijian, Russian, and American.

^d^Reason for participation: participants had the opportunity to select multiple reasons for participating in the trial. Other—hallucinations and dissociation identity disorder (n=1), anxiety and panic attacks (n=3), family issues (n=1), attention-deficit or hyperactivity disorder (n=1), and interest in marketing research (n=1).

^e^Not applicable.

### Acceptability

Three key themes emerged from the analysis of user and buddy feedback: (1) appeal of app features and layout, (2) usefulness of content, and (3) technological challenges. Although most users and buddies found Village appealing to use and easy to navigate, a few experienced difficulties downloading or using the app for the first time. This was particularly an issue for buddies who needed to be invited by users and did not have access to support from one of the research team (HK). Both users and buddies found Village helpful in constructing useful messages. However, some who had prior experience with mental health services found guided responses “robotic” and said they would prefer greater freedom to compose more personally relevant messages. The themes, subthemes, and supporting examples are summarized in Table 3. Most of the recruited participants (21/26, 81%) were retained in the study and completed the follow-up outcome measures.

**Table 3:** Young people and Buddy feedback on acceptability of Village app.

Theme and subthemes			Examples
**Appeal of app features and layout**			
	Ease of use	“I actually it was really straight forward, which is nice, like a lot of, I’ve tried other mental health apps before and a lot of them they’re either they’re really complicated to figure out or they’re just not intuitive but it felt like Village was quite intuitive, like it was kind of obvious what would, what buttons would do what.” (User 26) “I really enjoyed the experience of using Village. I felt that it was a really nice missing piece of interactions with a young person who’s struggling to be able to interact in that way.” (Buddy 26, friend)	
	Orientation challenges	“It was kind of confusing to get around, especially. Like if the, it’s not like a tutorial on how to use it. You kind of just turn right into it and like had no idea of what to do initially.” (User 23)	
**Usefulness of content**			
	Support with messaging	“I really enjoyed how the, like to explain how you’re feeling, they had the options for like what you could say. It made it easier because I know a lot of people, and especially me when I’m feeling really down, I find it really hard to know what to like to say to get help so I thought that was really good and helpful.” (User 11) “I looked at the tips and I read those and actually thought they were really, really good. They’re quite similar to, you know, what I learn at DBT^a^. I especially wanted to congratulate you on the validation portion. I think that that part was really good, really well written and absolutely perfect for the app.” (User 8) “You don’t have to think about what to write when you’re talking to a buddy, which is great because that’s a really annoying thing, having to think about what to say or trying to reach out and having no idea how to say it. I also liked the option to also edit that and put in your own things as well, your own thoughts. The hardest thing for me is knowing what to say when you need to ask for help or how to tell people how you’re feeling when you’re not feeling great. I think that made me feel a bit more confident on sharing how I’m feeling with someone and that’s definitely changed a lot about how I think about myself and accepting how I feel as well.” (User 17) “I can remember the tips that were saying, you know, you could respond like this or, you know, don’t forget to say this. And it was like, oh yeah that’s right, yep no I need to do that. So, they were quite useful to help compose a response.” (Buddy 1, parent)	
	Improved mental health knowledge	“I found the Discover section the most useful with the information. I thought the way that it was in snippets and then let you read more and stuff like that, it made it kind of seem like everything was backed up well by research and all that, which I thought was, you know, adds like an extra level of confidence to it. Gave me some insight on how to kind of talk to people who might be going through, you know, depression or anxiety, and that sort of thing.” (Buddy 11, friend) “It was pretty good actually, as well as interesting because like it kind of helped me get a bearing of what’s best to support people with those like common mental health issues, even if it wasn’t what the person I was supporting was dealing with.” (Buddy 15, friend) “I thought it was like a really good option because I know like, you know, lots of people don’t have a wide range of knowledge on different mental health issues so I thought that was like good, yeah informing me on issues.” (Buddy 21, friend)	
	Rote nature of responses	“It felt kind of, kind of clinical and removed though. Just with the way the responses were formulated.” (Buddy 7, friend) “I’m not sure how useful it was because he mentioned that it was like canned responses that he wasn’t able to like add much.” (Buddy 26, friend)	
	Positive impact on relationships	“I think it made a big difference. I went from like not talking to my friend, like anything like mental health wise to like actually like being able to like open up to them a lot more. I felt really like cared for and supported. Before, like I started using Village, I definitely if someone was to bring something up in real life, like I would shut it down, like the conversation or joke about it and try and move on. But it definitely, I felt a lot more comfortable, I wanted to talk about it, like in face-to-face if something was wrong.” (User 3) “I feel like my dad kind of he can sort of validate much better now because like before he was very like, it was like he was reading off a script every time. But with the app he’s discovered there’s so many other different ways to validate and I feel like he’s putting that into like real life and actually thinking about it, which has been really good.” (User 1)	
Technological issues			“There was a little hitch up with the signup but once we got around that it was, yeah smooth sailing.” (Buddy 11, friend) “The only kind of thing that kind of sucked was how you only get notifications whilst the app was open and not when it was closed.” (User 16) “Another one I found issue with in the app is friend requests. It was really confusing.” (User 23) “Occasionally actually it would log me out. I’m not sure why but sometimes I get logged out and have to relog in but that’s just a small thing.” (User 17)
Suggestions for improvement			“I think there was a few things that could have been improved like the avatar. There wasn’t too much of a variety to choose from.” [user 9] “Maybe having your phone receive notifications when the app’s closed as well.” (User 16)

^a^DBT: dialectical behavioral therapy.

### Feasibility

Overall, recruitment via social media advertising (n=14) was substantially more effective than recruitment via specialist mental health services (n=8) or primary health services (n=4). However, recruitment rates were reversed between these methods, with 80% (4/5) of those from primary care, 67% (8/12) of those from specialist services, and 5% (14/280) of those who heard about the study via social media actually being enrolled. This suggests that more effective collaboration with clinicians may be necessary for successful recruitment via clinical services. The relatively low uptake via all means among males might indicate that this group needs to be specifically targeted during the next phase of research. There were no reported issues with comprehension or completion of outcome measures using the REDCap software. Furthermore, no other unanticipated operational issues were observed.

### Usability

Usability of the Village app was evaluated using all 3 subscales of the uMARS questionnaire. Users gave Village a mean rating of 3.8 (range 2.7-4.6) out of 5 on a 5-point scale for app quality and an overall star rating of 3.4 out of 5 for subjective quality. Although knowledge, attitudes, and behavior change were rated lower (2.7-2.9 out of 5), the majority said it would increase the likelihood of future help-seeking (3.9 out of 5). Further details are provided in Table 4. Users reported varied engagement with Village during the trial. Some used the app multiple times a week, others occasionally, and a few only during COVID-19–related lockdowns. Examples of the feedback are presented in Textbox 1.

**Table 4:** Young people’s quality and impact rating of Village app using the Mobile Application Rating Scale^a^.

Rated item			Score, mean (SD)
**App quality rating**			
	Engagement (fun, interesting, customizable, interactive, and has prompts)	3.6 (0.6)	
	Functionality (app functioning, easy to learn, navigation, flow logic, and gestural design of the app)	4.0 (0.6)	
	Esthetics (graphic design, overall visual appeal, color scheme and stylistic consistency)	3.7 (0.7)	
	Information (contains high-quality information from a credible source)	3.8 (0.8)	
Total app quality mean score			3.8 (0.8)
**App subjective quality**			
	Would you recommend this app to people who might benefit from it? (1=not at all, 5=definitely)	3.0 (1.1)	
	How many times do you think you would use this app in the next 12 month if it was relevant to you? (1=none, 2=1−2, 3=3−10, 4=10−50, and 5≥50)	3.6 (0.9)	
	Would you pay for this app? (1=definitely not, 5=definitely yes)	1.6 (0.7)	
	What is your overall (star) rating of the app? (1=one of the worst apps I have used, 3=average, and 5=one of the best apps I have used)	3.4 (0.9)	
**App perceived impact**			
	Awareness (this app has increased my awareness of the importance of addressing the health behavior)	3.5 (1.4)	
	Knowledge (this app has increased my knowledge or understanding of the health behavior)	2.9 (1.1)	
	Attitude (the app has changed my attitudes toward improving this health behavior)	2.7 (1.1)	
	Intention to change (the app has increased my intentions or motivation to address this health behavior)	3.4 (1.2)	
	Help-seeking (this app would encourage me to seek further help to address the health behavior)	3.9 (1.1)	
	Behavior change (use of this app will increase or decrease the health behavior)	2.9 (1.0)	

^a^All rating scales ranged from 1 to 5 in the user version of the Mobile Application Rating Scale questionnaire.

**Examples of user feedback regarding pattern of app use.**

- “I used it when I was feeling bad and when I was feeling like I wanted to help my friends and stuff. So, I would go on the app to check if they needed any help or anything.” (User 16)
- “Most of the time I let people know when I wasn’t feeling good but like when I was overwhelmed more and when I wasn’t seeing my friends as much, when I wasn’t being able to check in on them in person. One of my friends I was messaging him probably like more, maybe like five times a week, especially like during the holidays and stuff when I wasn’t seeing him. And then other one µm like less often, maybe two times a week. And the other one maybe once a week.” (User 21)
- “If I’m being honest, at first, I didn’t really use it, but then, um, towards like the middle of like the trial, me and my friend would use it more because like lockdown and stuff happened and then there was another way to contact each other and let us know how we feel.” (User 11)

### Safety

The risk detection software was activated on 3 occasions during the study. Two followed users entering the words “burden” or “suicidal ideation” and one followed a buddy entering of the words “useless” and “not good enough” within the same message. On all 3 occasions, participants elected to be contacted by a member of the research team rather than being put through to a national telephone helpline, and they reported that they were fine. None of the participants interviewed toward the end of the study reported any episodes of self-harm or hospitalization during the period of enrollment.

### Efficacy

Preliminary evaluation of the efficacy of Village was undertaken by measuring changes in symptoms of depression, level of suicidal ideation, and level of functioning following app use; the results are presented in Table 5. In a repeated measures analysis, there was a statistically significant reduction in depression symptoms using the Patient Health Questionnaire–9 modified for adolescents between baseline and the 3-month follow-up (*P*=.002), but there were no statistically significant changes over time in suicidal ideation using the SIQ (*P*=.61) or in functioning using the WHODAS 2.0 or WHODAS-CY (*P*=.13). The severity scores at all time points for the 3 measures are displayed as tables in Multimedia Appendix 3.

**Table 5:** Changes in severity of depression, suicidal ideation, and functioning.

	Depression (PHQ-A^a^)				Suicidal ideation (SIQ^b^)				Functioning (WHODAS 2.0^c^ or WHODAS-CY^d^)		
	Baseline	4-week follow-up	3-month follow-up	Baseline		4-week follow-up	3-month follow-up	Baseline		4-week follow-up	3-month follow-up
Participant, n	26	18	21	26		20	21	25		19	20
Mean (SD)	18.1 (5.6)	15.7 (6.6)	12.7 (5.7)	77.7 (48.4)		68.9 (45.8)	57.3 (44.2)	46.2 (16.5)		45.2 (16.3)	36.7 (19.4)
Range	6.0-27.0	6.0-27.0	3.0-27.0	0-159.0		3.0-148.0	3.0-180	18.5-73.6		22.2-75.4	13.9-98.6
Mean difference (95% CI)^e^	N/A^f^	−2.4 (−6.3 to 0.9)	−5.4 (−9.2 to −2.2)	N/A		−8.8 (−42.7 to 13.2)	−20.4 (−53.9 to 1.2)	N/A		−1.0 (−13.2 to 8.9)	−9.5 (−21.7 to 0.2)
*P* value	N/A	.14	<.001	N/A		.29	.06	N/A		.69	.05
Effect size (Cohen *d*)^g^	N/A	N/A	0.9	N/A		N/A	0.4	N/A		N/A	0.5
Overall *P* value	N/A	N/A	.001	N/A		N/A	.16	N/A		N/A	.13

^a^PHQ-A: Patient Health Questionnaire–9 modified for adolescents.

^b^SIQ: Suicidal Ideation Questionnaire.

^c^WHODAS 2.0: World Health Organization Disability Assessment Schedule 2.0.

^d^WHODAS-CY: World Health Organization Disability Assessment Schedule–Children and Youth.

^e^Estimated marginal mean differences from a repeated measures analysis with pairwise comparisons between time points.

^f^N/A: not applicable.

^g^Effect sizes were calculated using baseline and 3-month follow-up means and SDs.

## Discussion

### Principal Findings

During this open trial, we found that Village was acceptable and usable to both users and buddies, and that a larger RCT appeared feasible, providing the following changes were made to the current version of the app and study protocol: (1) improved app onboarding instruction and notifications, (2) recruitment focused either directly on potential participants via social media or via collaborative clinicians at clinical services, and (3) all outcome measures being made mandatory on REDCap. Young people who experienced preexisting difficulty communicating with available support in person or via social media and buddies (families or friends) with limited mental health knowledge found the app most useful. The feedback suggested that some improvements to onboarding and notifications would further increase the appeal of the app. Short-term, statistically significant improvements in mood and nonsignificant changes in functioning or suicidal ideation need to be interpreted with caution given the small number of participants and the preliminary nature of the trial. Embedded risk detection software was appropriately activated on a few occasions and there were no reported episodes of self-harm or hospitalization among participants, suggesting the Village was safe to use with a clinically “high risk” cohort.

Although there is a plethora of digital interventions for supporting young people’s well-being and mental health, only a handful of other digital interventions have been specifically developed for young people experiencing self-harm and suicidal ideation. These are As Safe As Possible, during which young people complete a 3-hour safety-planning, emotion regulation session and then use an app to review their mood, safety plan, and learned skills [44]; Reframe-IT, a web-based cognitive behavioral therapy (CBT) program with 8 modules, video diaries, fact sheets, and a message board to interact with trained therapists [45]; and a Crisis Care mobile app that includes personalized coping skills accessible to both young people and parents [46]. Similar to Village, all 3 interventions have been piloted and shown to be acceptable to users, but not to improve mood, reduce self-harm, or reduce suicidal ideation. Better-powered trials of these interventions have been recommended [47], and until then, uncertainty regarding their efficacy remains. A larger number of nondigital family and peer support interventions have also been developed over the past decade. These include DBT, a manualized, multisession face-to-face therapy that includes young people and parents [48]; Safe Alternatives for Teens and Youth, a 12-week family-inclusive intervention based on principles of CBT and DBT [49]; the Family Intervention for Suicide Prevention, a single-session family intervention with telephone follow-up [50]; Attachment-Based Family Therapy, a therapy that aims to improve relationships between young people and caregivers and improve mental health symptoms [51]; Self-Harm Intervention Family Therapy, a 6-month family therapy-based treatment [52]; and integrated CBT, a combination of individual- and family-based CBT and parent training [53]. Of these, DBT had the strongest evidence (via 2 RCTs) of reducing self-harm and suicidal ideation in the short term, and youth-nominated support teams–version 2 [30] was the only intervention with evidence of long-term reduction of suicide. Others have demonstrated reasonable acceptability and variable short-term efficacy. Given the fluctuating nature of mood disorders and suicidal ideation, the timing and nature of outcome measurements are likely to be important in accurately evaluating the short-term efficacy of these interventions. Adequately powered studies and the collection of long-term follow-up data, such as those undertaken by King et al [30], are also essential and should be supported by funders.

Given that most young people these days regularly use texting and multiple social media apps to keep in touch with family and friends, it is reasonable to wonder about the necessity for a new vehicle with which to communicate distress. Previous research has shown that although social media can make it easier to maintain contact with others, young people are very careful about how to use such networks for support [54,55]. Concerns about public perception coupled with the need for trust and genuine understanding often lead those experiencing distress to be less open about their issues and perpetuate “false selves” [55]. Negative social media experiences make young people even less likely to use them when distressed [56]. When young people reach out via social media or in person, families and friends may not feel confident in responding because of their own feelings of discomfort, historically poor communication, or concerns about accountability [57,58]. Alternatively, they may respond in a manner that fails to address key emotions, thereby causing young people to feel no better or worse. Village affords a private forum for users to connect with chosen individuals in a genuine manner and for supporters to be educated about communication skills and mental health issues in a manner that leads them to sensitively respond to user needs. These factors are likely to increase both the depth of disclosure and satisfaction after disclosure [59].

Most young people in this trial chose friends, rather than family members, as buddies. This is consistent with previously identified age-related preferences for support among young people with depression [60]. It is also in keeping with developmentally related preferences identified during the co-design process for Village—younger adolescents (aged 13-16 years) who spent more time with their parents said they would prefer family members as buddies, whereas more independent and older adolescents (aged ≥16 years) said they would prefer to be supported by friends. Unfortunately, owing to ethical committee restrictions, younger adolescents were not included in this trial. However, we plan to include them in the upcoming RCT and conduct a subgroup analysis of acceptability to parents and friends to ensure that the app is useful to both groups.

Although conceptualized by an individual of Māori descent (ES), consciously designed to enhance “Whanau Ora,” and co-designed with a number of Māori young people and families (“whanau”), we did not conduct any specific evaluation of the cultural appeal or safety of the app during this open trial. Given the higher rates of depression, self-harm, and suicide among New Zealand Māori young people (consistent with rates among Indigenous groups in other countries [61]) and the concern that less-than-ideally designed digital interventions may increase health inequity for Indigenous populations [62], it is important for these issues to be explored before it can be assumed that the app is effective for those with the greatest need. Despite additional concerns regarding the potential for digital interventions to increase inequity between audiences with and without access to technology, we believe this is less of an issue in New Zealand where 95% of young people have internet access and 72% own a smartphone [63].

Despite the functionality of Village being rated above average, uMARS ratings were comparable with other recently developed mental health apps for young people [64], and despite users reporting improved awareness and greater likelihood of help-seeking following app use, it did not appeal to everyone. Onboarding instructions were not clear enough for everyone and were updated in light of participant feedback. Users and buddies with greater communication skills and mental health knowledge found the scaffolded communication restrictive. The pattern of app use varied considerably between participants, and most did not wish to use the app long term. It is possible that the ideal audience for the Village consists of young people who are relatively new to clinical services and family or friends with limited experience in providing mental health support. It is also possible that both audiences may learn what they need via the app and generalize communication or support skills to real life or other internet-based environments within a limited period. Village may play a vital role in “bridging the gap” between those in need and those who are able to access saturated mental health services [65]. Further research is required to investigate these possibilities.

The strengths of this trial include the exploration of both user and buddy perspectives on the app and the combined use of quantitative and qualitative analyses to provide a richer understanding of its appeal and function. Weaknesses include the limited sample size, lack of a control group, and lack of complete follow-up measures for 20% of participants, which might have led to biased results. In addition, not all users and buddies agreed to be interviewed, and interviews were conducted by a member of the research team who had been involved in participant recruitment and data analysis, and objective data regarding app use were not collected for privacy reasons. The exclusion of participants aged <16 years (owing to ethics committee constraints) and those from outside New Zealand also means that our results may not be generalizable to younger users or those from other countries.

### Conclusions

On the basis of the preliminary results from this open trial, Village appears to be an acceptable, usable, and safe communication app with which young people experiencing low mood, self-harm, and suicidal ideation can receive support from their family and friends. A larger RCT to confirm the current findings and evaluate the efficacy of the app appears to be feasible with minor modifications to the app and study protocol.

## Data Availability

Data are presented in the multimedia appendices.

## Appendix

Multimedia Appendix 1 Higher resolution version of Figure 2. Supported messages.

Multimedia Appendix 2 Semistructured interview questions.

Multimedia Appendix 3 Severity scores for depression, suicidal ideation, and functioning.

## Abbreviations

CBT: cognitive behavioral therapy
DBT: dialectical behavioral therapy
RCT: randomized controlled trial
REDCap: Research Electronic Data Capture
SIQ: Suicidal Ideation Questionnaire
uMARS: user version of the Mobile Application Rating Scale
WHODAS: World Health Organization Disability Assessment Schedule
WHODAS-CY: World Health Organization Disability Assessment Schedule–Children and Youth
