# Supplementary figures and images for: Acceptability and Feasibility of “Village,” a Digital Communication App for Young People Experiencing Low Mood, Thoughts of Self-harm, and Suicidal Ideation to Obtain Support From Family and Friends: Mixed Methods Pilot Open Trial
Source: JMIR Form Res. 2023 Mar 13;7:e41273. doi: 10.2196/41273 (PMC10131861; doi:10.2196/41273)

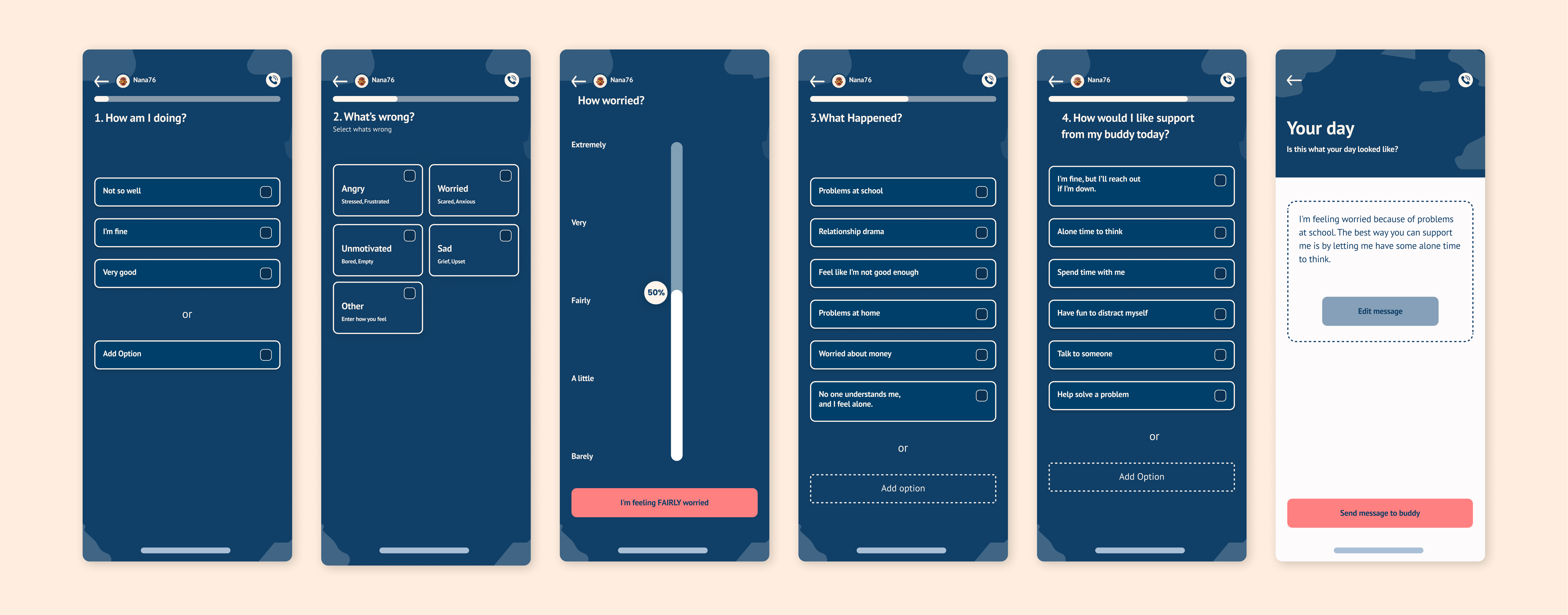

Supplement: Multimedia Appendix 1 [file formative_v7i1e41273_app1.png]
