# Supplementary material for: Acceptability and Feasibility of “Village,” a Digital Communication App for Young People Experiencing Low Mood, Thoughts of Self-harm, and Suicidal Ideation to Obtain Support From Family and Friends: Mixed Methods Pilot Open Trial
Source: JMIR Form Res. 2023 Mar 13;7:e41273. doi: 10.2196/41273 (PMC10131861; doi:10.2196/41273)
Supplement: Multimedia Appendix 2 [file formative_v7i1e41273_app2.docx]

# Appendix 2: Semi-structured interview questions

# Introduction for both types of participant:

Thank you for agreeing to answer some questions about your experiences of the Village app. Please be as honest as you can about your experiences. We hope to use the information that you provide us to improve the app before it is used by other people. We will record your responses so that we can accurately remember what you have told us. We will combine your responses with those from other study participants in order to identify key themes. You will not be personally identified via the information you provide.

# Questions for young people:

1. Can you tell us how it was to use the app?
2. We’d like to ask you some questions about the look and feel of the app:
   - What did you think about the app’s appearance and design?
   - To whom do you think the app would mostly appeal? If not you, why not?
   - How could the look and feel of the app be improved to better suit you?
3. We’d now like to ask you some questions about the technological features of the app:
   - How easy was it to use the app?
   - Did you experience any technological issues while using it? (If so, tell us more about it)
   - How did you find navigating between the different dashboards on the app?
   - How could we improve the technology within the app?
4. We’d also like to ask you some questions about how you used the app:
   - How often did you use the app? Were there any particular times when you used it? (feeling low, sad or stressed, the daily reminders?)
   - How many buddies did you choose/identify?
   - What is your relationship to the buddies you choose to invite on the app?
   - How often did you interact with your buddies? Was it the same or different for different buddies?
   - How useful did you find contact to/from your buddies?
   - How useful did you find the daily reminders?
   - Tell us what other features of the app you used?
   - Did you use/what did you think of the option-guided and emotion-rating messaging feature?
   - Tell us more about what you found most useful or least useful about the app
   - What difference do you think using the app made to your life over the past month? How well did you feel supported by your buddies during this time?
   - How do you think the app may have been useful for your buddies? Was this the same for all of them? Did they give you any feedback about communicating via the app? Did communication via the app change any of the way that they communicated with you in real life?
5. Did you have any other worries or encounter any problems while using the app?
6. Are there any improvements we can make to the tips for you or your buddies on the app? (What kind of tips or guidance would be beneficial?)
7. Can you think of any other ways in which we can improve the app?
8. How likely are you to keep the app on your device?
9. Finally, is there anything else you’d like to tell us about your experience

# Questions for buddies:

1. Can you tell us how it was to use the app?
2. We’d like to ask you some questions about the look and feel of the app:
   - What did you think about the app’s appearance and design?
   - To whom do you think the app would mostly appeal? If not you, why not?
   - How could the look and feel of the app be improved to better suit you?
3. We’d now like to ask you some questions about the technological features of the app:
   - How easy was it to use the app?
   - Did you experience any technological issues while using it? (If so, tell us more about it)
   - How did you find navigating between the different dashboards on the app?
   - How could we improve the technology within the app?
4. We’d also like to ask you some questions about how you used the app:
   - How often did you use the app? Were daily reminders useful to check in on your young person?
   - How often did you interact with your young person? Tell us more about this.
   - How useful did you find contact to/from your young person? Tell us more about this.
   - How useful did you find information about common mental health problems and how to communicate with/support young people? Tell us how you used this information.
   - Tell us what other features of the app you used.
   - Tell us more about what you found most useful or least useful about the app
   - What difference do you think using the app made to both you and your young person over the past month?
   - Did communication via the app change any of the way that you communicated with your young person in real life?
5. Did you have any other worries or encounter any problems while using the app?
6. Are there any improvements we can make to the tips for buddies on the app? (What kind of tips or guidance would be beneficial?)
7. Can you think of any ways in which we can improve the app?
8. Finally, is there anything else you’d like to tell us about your experience?
