# Supplementary material for: Acceptability and Feasibility of “Village,” a Digital Communication App for Young People Experiencing Low Mood, Thoughts of Self-harm, and Suicidal Ideation to Obtain Support From Family and Friends: Mixed Methods Pilot Open Trial
Source: JMIR Form Res. 2023 Mar 13;7:e41273. doi: 10.2196/41273 (PMC10131861; doi:10.2196/41273)
Supplement: Multimedia Appendix 3 [file formative_v7i1e41273_app3.docx]

**Appendix 3: Severity scores for depression, suicidal ideation and functioning**

**Depression severity scores**

| **ID** | **Baseline** | **4 weeks** | **3 months** |
| --- | --- | --- | --- |
| **01** | 26 | 23 | 15 |
| **02** | 23 | 23 | 19 |
| **03** | 26 | 10 | 6 |
| **04** | 19 | - | 15 |
| **05** | 13 | 13 | 10 |
| **06** | 10 | 10 | 8 |
| **07** | 22 | 20 | 18 |
| **08** | 22 | 18 | 15 |
| **09** | 20 | - | 6 |
| **10** | 20 | 11 | 11 |
| **11** | 15 | 13 | 13 |
| **12** | 27 | 27 | 27 |
| **16** | 15 | 11 | 10 |
| **17** | 24 | 27 | 17 |
| **19** | 19 | 21 | 13 |
| **21** | 18 | 18 | 21 |
| **22** | 6 | = | 13 |
| **23** | 14 | 13 | 11 |
| **24** | 14 | 11 | 3 |
| **26** | 15 | 7 | 6 |
| **27** | 13 | 6 | 9 |
| **37** | 20 | - | - |
| **30** | 26 | - | - |
| **31** | 23 | - | - |
| **14** | 10 | - | - |
| **28** | 18 | - | - |

**Suicidal Ideation severity scores**

| **ID** | **Baseline** | **4 weeks** | **3 months** |
| --- | --- | --- | --- |
| **01** | 148 | 135 | 92 |
| **02** | 159 | 135 | 130 |
| **03** | 65 | 39 | 33 |
| **04** | 94 | - | 75 |
| **05** | 16 | 17 | 13 |
| **06** | 53 | 43 | 40 |
| **07** | 119 | 98 | 106 |
| **08** | 137 | 127 | 116 |
| **09** | 66 | 42 | 23 |
| **10** | 54 | 84 | 43 |
| **11** | 33 | 38 | 29 |
| **12** | 156 | 131 | 180 |
| **16** | 112 | 38 | 39 |
| **17** | 109 | 148 | 48 |
| **19** | 56 | 63 | 66 |
| **21** | 21 | 42 | 40 |
| **22** | 60 | 30 | 35 |
| **23** | 38 | 31 | 30 |
| **24** | 103 | 96 | 37 |
| **26** | 32 | 38 | 25 |
| **27** | 0 | 3 | 3 |
| **37** | 67 | - | - |
| **30** | 174 | - | - |
| **31** | 145 | - | - |
| **14** | 46 | - | - |
| **28** | 112 | - | - |

**Functioning severity scores**

| **ID** | **Baseline** | **4 weeks** | **3 months** |
| --- | --- | --- | --- |
| **01** | 43.1 | 25.5 | 20.5 |
| **02** | 67.6 | 75.4 | 58.5 |
| **03** | 73.6 | 44.8 | 30.4 |
| **04** | 37.5 | - | 24.8 |
| **05** | 23.3 | 27.6 | 33.6 |
| **06** | 50.3 | 22.2 | 17.8 |
| **07** | 26.1 | 73.3 | 51.2 |
| **08** | 66.5 | 49.8 | 44.7 |
| **09** | 44.9 | 38.2 | 18.5 |
| **10** | 54.6 | 48.5 | 28.5 |
| **11** | 52.4 | 41.0 | 42.4 |
| **12** | 71.1 | 62.9 | 98.6 |
| **16** | 33.8 | 27.3 | 34.8 |
| **17** | 57.1 | 67.6 | 40.5 |
| **19** | 47.3 | 54.2 | 54.2 |
| **21** | 37.1 | 42.0 | 44.5 |
| **22** | 39.9 | 38.3 | 13.9 |
| **23** | 18.5 | 32.1 | 23.2 |
| **24** | 56.5 | 57.2 | 24.5 |
| **26** | 23.3 | 31.5 | 28.2 |
| **27** | **-** | - | - |
| **37** | 59.4 | - | - |
| **30** | 86.9 | - | - |
| **31** | 63.4 | - | - |
| **14** | 32.4 | - | - |
| **28** | 18.8 | - | - |
